# Situated Neural Representations: Solving the Problems of Content

**DOI:** 10.3389/fnbot.2022.846979

**Published:** 2022-04-14

**Authors:** Gualtiero Piccinini

**Affiliations:** Department of Philosophy and Center for Neurodynamics, University of Missouri—St. Louis, St. Louis, MO, United States

**Keywords:** neural representation, neural computation, semantic content, situated cognition, embodiment, embeddedness, enactivism, affect

## Abstract

Situated approaches to cognition maintain that cognition is embodied, embedded, enactive, and affective (and extended, but that is not relevant here). Situated approaches are often pitched as alternatives to computational and representational approaches, according to which cognition is computation over representations. I argue that, far from being opposites, situatedness and neural representation are more deeply intertwined than anyone suspected. To show this, I introduce a neurocomputational account of cognition that relies on neural representations. I argue not only that this account is compatible with (non-question-begging) situated approaches, but also that it *requires* embodiment, embeddedness, enaction, and affect at its very core. That is, constructing neural representations and their semantic content, and learning computational processes appropriate for their content, requires a tight dynamic interaction between nervous system, body, and environment. Most importantly, I argue that situatedness is needed to give a satisfactory account of neural representation: neurocognitive systems that are embodied, embedded, affective, dynamically interact with their environment, and use feedback from their interaction to shape their own representations and computations (1) can construct neural representations with original semantic content, (2) their neural vehicles and the way they are processed are automatically coordinated with their content, (3) such content is causally efficacious, (4) is determinate enough for the system's purposes, (5) represents the distal stimulus, and (6) can misrepresent. This proposal hints at what is needed to build artifacts with some of the basic cognitive capacities possessed by neurocognitive systems.

## The Problems of Content

Explaining cognition in terms of neural computations over neural representations, as mainstream cognitive neuroscience does, raises tough foundational questions. Among the most difficult are a cluster of related problems pertaining to the putative semantic content of neural representations. I will refer to them as the *problems of content*:

1. *The source of original content* (cf. Haugeland, 1998; Jacob, 2019). The semantic content of public language and other public symbolic systems is *derivative*—that is, it seems to derive from other entities, the symbols' users, whose states appear to possess semantic content of their own. For instance, the word “burro” means *butter* in Italian but *donkey* in Spanish; the very same physical symbol—the same sequence of phonemes or letters—can mean different things in different languages. The most plausible explanation is that the content of words such as “burro” derives from the speakers of the different languages within which the words occur. In contrast, if intelligent agents operate via representations internal to their neurocognitive systems, the semantic content of their internal representations must be *original*—it cannot be derived from other semantically contentful sources on pain of vicious regress. But there is no consensus on how neurocognitive states can acquire original semantic content.
2. *The coordination between vehicles and their content* (cf. Fodor, 1994, p. 12ff, 86; Piccinini, 2004, p. 405). Representational explanation requires that vehicles be processed computationally in a way that matches their content. For example, suppose we want a computer to perform inferences about animals. The inferences the computer performs must match the meaning of its various symbols: for instances, from “there is a dog” the computer may infer “there is a barking animal” but may not infer “there is a meowing animal”; the opposite must hold for “there is a cat.” In ordinary artificial computers, the match between computations and the semantic content of the vehicles is accomplished by the programmer, who can independently access both the computational vehicles and their content and program the computer accordingly. In the case of neurocognitive systems, however, there is no external programmer. Thus, it is unclear how computational vehicles and the computations performed over them can be matched to appropriate semantic contents. It seems that any putative mechanism tasked with matching vehicles and the way they are processed to the vehicles' semantic content must have independent access to both vehicles and their contents, so that it can match them accordingly. This would require that vehicles and contents be accessible independently of one another within the neurocognitive system, which does not seem possible.
3. *The causal efficacy of content* (cf. Stich, 1983; Dretske, 1988; Fodor, 1994). Insofar as representations explain behavior, they appear to do so in virtue of their content. For instance, suppose that my dog Cinnamon licks my face because she is happy that I'm back home, and this is cashed out in part in terms of Cinnamon's neural representation whose semantic content is that *I'm back home*. Such semantic content is supposed to contribute to the explanatory power of representations. For, if Cinnamon's representation, causing her to lick my face, had a different content—e.g., *that the cat is meowing*—then a representational explanation of why Cinnamon is licking my face would fail. But what causes behavior is the vehicle that carries the content, which is what the system physically processes. Since the causal work is done by the vehicle, the semantic content has no causal work left to perform. In addition, semantic content appears to be relational in a way that undermines its causal efficacy. For semantic content is a relation between the vehicle and what it represents, and that does not seem to be the sort of thing that can play a causal role. If these observations are correct, then semantic content plays no causal role. If so, content is epiphenomenal and representational explanation is illusory. The genuine explanation of behavior is causal and, therefore, it can't appeal to semantic content.
4. *The determinacy of content* (cf. Shea, 2018; Neander and Schulte, 2021). It seems to many that a notion of representation worthy of its name should come with determinate semantic content—the kind that can be expressed by a proposition and evaluated as true or false or, in the case of concept-like representations, the kind that can be expressed by a linguistic predicate. But theorists disagree about what content neural representations have. A classic example is what the frog's eye tells the frog's brain (Lettvin et al., 1959). Even theorists who agree pretty closely on what determines the semantic content of neural representations have offered different interpretations of the internal signals that allow frogs to detect, catch, and eat bugs. They have proposed that the signals' content is (i) *fly (there now)*, (ii) *something small, dark, and moving (there now)*, or (iii) *food (there now)*. There is no consensus on how to resolve this disagreement. This suggests that putative neural representations lack determinate contents after all, which in turn suggests that neural vehicles are not representations properly so called.
5. *The distality of content* (e.g., Dretske, 1988; Shea, 2018; Neander and Schulte, 2021). Between a stimulus and a neural state, there is a causal chain involving many intermediate causes, all of which correlate with the internal state and all of which may be said to cause the internal state. For instance, a visual stimulus such as a flower in a garden causes patterns of light waves that travel through the air, which cause activation patterns in the retinas, which cause spike trains to travel through the optic nerve, which cause activation patterns in the lateral geniculate nucleus of the thalamus, etc. Many naturalistic theories of content assign content at least in part based on the relation between a representation and what causes it (Adams and Aizawa, 2021). If the content of a representation is determined by what causes it, however, it's unclear why a neural state should represent the distal cause—e.g., the flower—rather than any of its more proximal causes.
6. *The possibility of misrepresentation* (e.g., Dretske, 1986; Fodor, 1994; Neander and Schulte, 2021). If a system can represent, it should also be able to *mis*represent. For instance, if visibility is poor, a system might mistake a horse for a cow, thus representing a horse as a cow. As noted above, however, many naturalistic theories of content appeal to the relation between a representation and what causes it. Accordingly, if a representation is caused by a horse, its representational content should be *horse*, not *cow*. But then it's unclear how a representation can ever misrepresent. There is no consensus about how a naturalistic theory of content can account for the possibility of misrepresentation.

The difficulty of one or more of these problems has led many theorists, including many theorists of situated cognition, to reject neural computations, neural representations, or both (e.g., Casper and Artese, 2020). According to such theorists of situated cognition, cognition is situated—that is, embodied, embedded, enactive, and affective—*as opposed to* representational and computational (e.g., Thompson, 2007). Many others continue to maintain that cognition involves computation over representation, and some have correctly pointed out that cognition being representational and computational is compatible with cognition being situated (e.g., Clark, 1997; Miłkowski, 2017). Even among such compatibilists, however, there is no consensus on how to fully solve the problems of content.

I will argue not only that situatedness is compatible with computation and representation, but also that the situatedness of neurocognitive systems and, as a consequence, *the situatedness of neural computations and representations is the very key to solving all the problems of content at once*. Specifically, I will argue that neurocognitive systems that are embodied, embedded, affective, dynamically interact with their environment, and use feedback from their interaction to shape their own representations and computations (1) can construct neural representations with original semantic content, (2) their neural vehicles and the way they are processed are automatically coordinated with their content, (3) such content is causally efficacious, (4) is determinate enough for the system's needs, (5) represents the distal stimulus, and (6) can misrepresent.

Caveat 1: The most successful account of the semantic content of internal representations is informational teleosemantics (Dretske, 1988; Neander, 2017; Shea, 2018). Roughly, according to informational teleosemantics, the semantic content of (indicative) representations is the information they have the function to carry. Existing versions of informational teleosemantics go at least part of the way toward solving problems 1 and 4-6. This is largely because teleosemantics already includes an important element of situatedness: the teleofunctions that give teleosemantics its name are *wide* functions—functions that reach into the organism's environment. That said, problems 2 and especially 3 are harder to crack; I will argue that solving them along with fully solving the others requires a more thorough appeal to the organism's situatedness. As I will point out, the recent literature contains hints that the solutions to the problems of content are to be found in the situatedness of neural representations. The considerations to follow are intended to (i) improve on existing versions of teleosemantics by (ii) making points that are either overlooked or only implicit in the teleosemantics literature, thereby (iii) showing how the situatedness of neurocognitive systems contributes to solving the problems of content and (iv) providing a unified solution to the problems of content.

Caveat 2: I will not propose a complete account of intentionality. For present purposes, intentionality is the property of indicative mental states, such as beliefs, to the effect that they can be attributed a propositional content with full-blown truth conditions, as opposed to the kind of accuracy conditions that I will adopt as a standard for the kind of (nonpropositional) neural representations that make up the bulk of the cognitive economy of most animal species. Explaining intentionality involves explaining fully determined propositional contents, referential opacity, representation of nonexistent objects, and other phenomena that go beyond the scope of this essay. What I will do is propose a solution to some of the most difficult problems faced by an account of basic neural representations with original semantic content, problems which lie at the foundation of any naturalistic theory of intentionality. Fully accounting for intentionality itself is a separate project, which will require additional work (for steps in that direction, see Morgan and Piccinini, 2018; Piccinini, 2020b).

Caveat 3: I will set phenomenal consciousness aside. The relationship between neural representation (and computation) and phenomenal consciousness is challenging territory that lies outside the scope of this paper (for more detailed discussion of options and some hints at the direction that appears most promising, see Piccinini, 2020a, Ch. 14 and Anderson and Piccinini, unpublished, Ch. 7).

Caveat 4: In addition to embodiment, embeddedness, enaction, and affect, situated approaches also include the thesis of extended cognition, that is, that some cognitive states or processes occur outside the skull. Whether cognition is extended does not affect my argument, so I will remain neutral about that.

## Basic Framework

I will adopt a theoretical framework defended in detail by Piccinini (2020a, 2022). Here I will briefly recap the main aspects that are relevant to this project. This section is intended primarily for philosophers; non-philosophers can skip it without too much loss.

The universe consists of many objects that stand in compositional relations: small objects compose larger objects, which compose ever larger objects until all objects, taken together, compose the whole universe. Objects have natural properties, including relational properties. There are three types of property: qualities, such as shape and size; causal powers, such as the ability to fire action potentials; and structural properties, such as being made of neurons and glial cells arranged in a certain way. An object's properties are invariant aspects of the properties of that object's parts. A composite object itself is an invariant under certain transformations in its parts.

The objects we are concerned with are substantive wholes, namely, objects whose (proper) parts change their properties when they come to stand in organizational relations such that the parts compose such wholes. For instance, pluralities of disconnected neurons and glial cells cannot perform nontrivial cognitive functions. When they are connected together and sustained by an organism's metabolism, however, neurons and glial cells form nervous systems, thereby acquiring the ability to send signal to one another and, collectively, to perform nontrivial cognitive functions.

It's important to note that causal powers require disposition partners for their manifestation and are typically individuated by the manifestations they have when they encounter their partners (Martin, 2008). For instance, the very notion of a *signal* presupposes that the signal is sent to one or more receivers. Accordingly, the power to send a neuronal signal presupposes a communication channel through which a neuron sends the signal to one or more receivers. Thus, causal powers include an intrinsic aspect—what the object can contribute to a manifestation—and a relational aspect—what the object must be related to in order for the manifestation to occur.

It's also important to note that a property can be both the manifestation of an object's causal powers as well as a causal power of its own. For instance, a truck's momentum is both a manifestation of its power to set itself in motion and a causal power of its own, which can be transferred to other objects in case of collision. Later I will argue that semantic content is a case of this sort: both the manifestation of some of a neurocognitive system's causal powers and a causal power of its own.

Some systems contain *mechanisms*. For present purposes, mechanisms are subsystems composed of different types of part, each with its own specialized powers, and the parts are organized in such ways that each part meets disposition partners in some of the mechanism's other parts and portions of the environment of the system. As a result, mechanisms have powers that their parts, when they are not organized to form the mechanism, could never have.

Some special mechanistic systems are *organisms*. What counts as an organism is a difficult question that I cannot address in depth here. Suffice it to say that organisms have special closure properties such that their parts are mutually involved in maintaining the organization of the system^1^. Organisms include sets of entities each of which can be produced from other entities within the set (Kauffman, 1993), organisms exert work to maintain internal constraints that in turn are necessary to produce the work (Kauffman, 2002), their processes are mutually constrained in such a way that each constraint is generated by at least one other constraint (Montévil and Mossio, 2015), and their behavior, broadly construed to include metabolism, must result at least sometimes in a mutually supportive set of conditions that include survival, development, reproduction, and helping others (Piccinini, 2020a, p. 68). I call the latter four conditions *goals* in the following minimal sense: they require work and, if all members of a population fail to fulfill them, eventually the population goes extinct. Thus, for organisms to continue to exist, the four goals must be pursued and fulfilled at least sometimes by some organisms.

Since organisms have goals that they must pursue, their traits (parts and their properties) as well as the artifacts they build and use may contribute to such goals. Contributing to such goals in a stable way is what I call the biological *function(s)* of such traits and artifacts. Token traits and artifacts that belong to a type some of whose tokens are able to perform a function may be said to have that function even though they cannot perform it or cannot perform it at the appropriate rate in appropriate situations. Thus, this is a normative notion of function: traits and artifacts can function incorrectly, malfunction, or completely fail to perform their function^2^.

Some organisms have specialized control organs—namely, nervous systems—whose function is to direct the behavior of the organism as a whole in response to environmental, physiological, and developmental conditions. Fulfilling control functions requires transducing different kinds of external signals into internal vehicles that allow the control organ to integrate different sources of information, build and update internal models of the body and environment, and use such models to guide and direct behavior. Since the function of the vehicle is to encode different sources of information as well as guide the control of a complex organism, the vehicles themselves are defined in terms of such functions, not any particular ways in which the vehicles are physically implemented. I call such vehicles medium-independent, and the manipulation of such vehicles in a rule-governed way, which is needed to perform control functions, *computation* in a generic sense. While neural processes are computational in a generic sense, there are good reasons to conclude that they are sui generis computations—neither digital nor analog^3^.

## Neural Structural Representation

There is a widespread consensus that the notion of representation that is relevant to cognitive neuroscience is that of *structural* representation^4^. To a first approximation, a structural representation is a model of a target that can guide behavior with respect to its target. For example, a map of a territory is a structural representation. More precisely, I define a structural representation as a system that has the function of possessing the following four features: (i) a partial isomorphism (homomorphism^5^) to its target, (ii) being activated by signals coming from its target, (iii) the ability to guide behavior with respect to its target, and (iv) the ability to be decoupled from signals coming from its target (and therefore to guide behavior with respect to its target even when its target is not directly activating the representation)^6^.

Ramsey argues that, in addition to defining structural representation in such functional terms (a model that can guide behavior), we also need an account of the semantic content of structural representation (Ramsey, 2007, 2016). He points out that many theorists either fail to distinguish between the functional role of structural representations and their semantic content or they simply ignore the functional role.^7^ Ramsey concludes that, in addition to an account of functional role along the lines I gave above, we also need an account of the representations' semantic content. The most successful account of the semantic content of structural representations is informational teleosemantics, which says, roughly, that the semantic content of a structural representation is the information it has the function of carrying about its target (Dretske, 1988; Neander, 2017; Shea, 2018). For present purposes, that a state carries information about a target means that the occurrence of that state raises the probability that the target is also occurring.

I agree that the notion of structural representation is the relevant one, and I will endorse a version of informational teleosemantics. I add that neural representations have special features such that, when the relevant notion of structural representation and the relevant teleosemantic theory are formulated properly, *the vehicles of neural representations and their semantic content are two sides of the same coin*. That is, the same functional properties that turn a system of internal states into a neural representational system are also sufficient to give such internal states their semantic content^8^. I will also argue that, once we gain an adequate account of the ontology of original semantic content, the content of neural representations is an aspect of their causal powers—the power to track their target and, as a consequence, to guide behavior with respect to their target.

For present purposes, a neural structural representation is a state of a *simulation* of a target, where a simulation is a system of states, homomorphic to their target, which can evolve to match the evolution of their target to some degree of approximation. In addition, a *neural* structural representation is a state of a system whose functions includes the following:

1. To build and maintain a simulation of its body and environment.
2. To use the simulation to guide behavior by issuing motor commands.
3. To use information from the body and environment together with its own motor commands to update the simulation.

A system that performs the above functions has all the four features of structural representations. By definition, the simulation it builds and maintains is homomorphic to its target and can guide behavior. By relying on information from the body and environment to update its internal states, the system gets activated by signals from its target. Finally, since the simulation is a dynamical model that can evolve on its own in a way that can match its target, its states can be decoupled from their target.

A system that performs the above functions already has all that's needed for its states to have semantic content according to informational teleosemantics^9^. This is because, since one of the system's functions is building a simulation of its environment and updating it using information from the environment, the states of the simulation carry information about environmental states. We may conclude that one of the states' functions is tracking their targets, or we may prefer to say that they track their target, when they do, due to the function of the system as a whole; regardless, this is enough for a viable teleosemantics. It is in virtue of the information they carry about their targets that such states can guide behavior with respect to their targets.

Now let's consider the metaphysics of the semantic content of this kind of structural representation. Recall from the previous section that causal powers include an intrinsic aspect—what the object can contribute to a manifestation—as well as a relational aspect—what the object must be related to in order for the manifestation to occur. Each state of the sort of simulation we are discussing has an intrinsic aspect—the ability to receive, process, and send signals—and a relational aspect—the relations to the rest of the system. It is the relations to the rest of the system, which in turn is related in appropriate ways to the body and environment, which enable each internal state to receive and send signals carrying information about their target and to guide behavior on that basis.

On one hand, the system has learned to activate each internal state to track specific targets and predict the target's evolution. Thus, when the system functions correctly, each internal state sends its signals under appropriate circumstances (information is flowing in either directly from the target or from other internal states that carry information about the target, including past states of the system). On the other hand, when the system functions correctly, each internal signal can be used to guide behavior in the relevant way—i.e., with respect to its target. As a result of the combination of its intrinsic and relational properties, each internal state has the causal power to track its target, predict the target's evolution, and guide behavior with respect to its target (to the extent that the system is performing its representational function)^10^.

The semantic content of a neural representation is a manifestation of its power to track its target and predict its evolution. It is also the causal power to guide behavior with regards to its target. Content is often represented by using that-clauses; for example, “the cat is on the mat” means *that the cat is on the mat*. This is an inadequate way of expressing the content of neural representations, at least in the general case. On one hand, typical neural representations do not enter the kind of explicit inferential relations that linguistic representations, whose content is expressed by that-clauses, can enter. In addition, their correctness conditions are a matter not of truth or falsehood but of degrees of accuracy with which a target is tracked. On the other hand, however, neural representations are rich in detail, connected to other representations, dynamical, and predictive of their target's evolution in a way that linguistic representations are not. Thus, the content of a neural representation of the cat being on the mat may be *very* roughly approximated as follows: *cat on mat there now and will likely evolve in such and such a way*. Notice that I didn't use a that-clause, because typical neural representations are not propositional representations but simulations of their target.

A specific content may be distributed over a relatively large ensemble of neurons. Yet content is relatively localized in the sense that it is carried by a specific vehicle born by a specific bearer (neuron/ensemble/circuit) and not diffused through the whole neurocognitive system, or even a large part thereof. Yet content (qua causal power) also depends on the causal role that the firing of a neuron/neural ensemble/neural circuit plays within the neurocognitive system, so it depends on the structural and functional relations between the vehicle (and therefore the vehicle bearer, the neuronal structure) and other relevant portions of the system. Since content is acquired by the neurocognitive system through learning via feedback from the environment (more on this below), it is acquired holistically thanks to the action of a system larger than the bearer of the content, and it depends on the holistic relations between its bearer and the rest of the system for its existence qua content. Yet content is also somewhat localized in the sense of being possessed by a small part of the system in virtue of the specific causal role that subsystem plays within the whole system.

In other words, the content of a neural representation is a manifestation of a causal power (the power to track a target), yet this content is created by a broader learning process involving a larger system, and the fact that it functions as content is made possible by the broader causal role that the content plays in guiding behavior within the system.

In summary, there are three causal processes pertaining to content: the learning process that creates the content, the causal process that defines the content (as tracking a certain target and predicting its evolution), and the causal process that makes it possible for the content to guide behavior.

## The Situatedness of Neural Representation

For neural representations to exist at all, the system that constructs and maintains them—the neurocognitive system—must be embodied, embedded, enactive, and affective. This situatedness of neural representations is needed because neural representations and the computations that are interdependent with them emerge diachronically through the dynamical interaction between the nervous system, its body, and its environment in a way that must take into consideration the organism's needs. Let's unpack this point, one step at a time.

Neurocognitive systems are made out of neurons and other cells; the neurons, connected into networks, are the main components performing cognitive functions. The structure and functions of neurocognitive systems are innately constrained. The structure and functions of an organism's body affect how its neurocognitive system develops and what processes it performs (Chiel and Beer, 1997). In addition, developmental processes that are at least partially under genetic control determine the differentiation of the neurocognitive system into different systems (cortex, cerebellum, hippocampus, etc.), the formation of different subsystems (cortical areas, columns, nuclei), much of the wiring between systems and subsystems, the main biophysical properties of different types of neurons, the transduction of external stimuli into firing rates within sensory systems, the transduction of firing rates into muscle contractions at the neuromuscular junction, and so forth. All these factors constrain the type of representations and computations neurocognitive systems can perform and the kinds of behaviors they can exhibit (e.g., Kim et al., 2017; Wang et al., 2018). That said, one of the most important features of neurocognitive systems, which is also built through development, is their *plasticity*, that is, their ability to change their structure and functions in response to their dynamic interaction with body and environment. Plasticity is the basis for the ability to *learn*, which in turn allows neurocognitive systems to construct and shape their representations and computations.

The study of how biological neural networks learn has influenced and has been influenced by the study of *artificial* neural networks. Comparing the types of learning that occurs in biological vs. artificial neural networks will help us highlight how important situatedness is to learning in biological neural networks and what might still be missing from current AI technology.

Artificial neural networks can learn in three main ways: supervised, unsupervised, and by reinforcement. *Supervised* learning occurs when an agent external to the network calculates the error produced by the network, uses such error to adjust the structure (and therefore the functions) of the network to improve performance, and repeats this process until the network exhibits the desired performance. This is often done by feeding the network labeled data during the training period, that is, inputs that already include information about how the network is supposed to classify the data. In contrast, *unsupervised* learning occurs when the network itself adjusts its structure (and therefore its functions) in response to its inputs in order to find, extract, and represent similarities, invariants, and associations within its inputs, without receiving external feedback on how to improve its performance. Finally, *reinforcement* learning occurs when a network performs actions in response to its input, receives a reward signal in response to successful actions, and uses the reward signal to adjust which actions it will select in the future (Sutton and Barto, 2020).

Both supervised and traditional unsupervised learning have limits. Supervised learning is limited by the requirement of labeled data, which may or may not be always available in large enough quantity. Unsupervised learning is limited by the absence of any external information on how the inputs should be processed; thus, it works best for tasks that require merely extracting patterns from inputs. To overcome these limitations, a more recent approach involves a type of unsupervised learning called *self-supervised* learning: artificial neural networks that learn by extracting supervisory signals from the data themselves, without relying on explicit labels supplied by external agents. By relying on the structure of the data, self-supervised learning networks attempt to predict one portion of an input from another portion, and then use any resultant discrepancy to improve their representations and computations. Adding the ability to learn from rewards and punishments turns a neural network into a *reinforcement* learning network, which allows it to learn by trial-and-error how to respond to different situations.

None of the training methods for artificial neural networks are a perfect fit for the type of learning that occurs within neurocognitive systems. Unlike in supervised learning, there are no external agents labeling the data that enter neurocognitive systems or calculating how the structure of neurocognitive systems should be adjusted to improve performance. Therefore, neurocognitive systems do not undergo supervised learning as it occurs in artificial neural networks. In addition, unlike traditional unsupervised learning, neurocognitive systems are not limited to processing their inputs in the absence of external feedback.

The types of AI learning that are closest to what neurocognitive systems do are self-supervised learning and reinforcement learning. Like artificial systems undergoing self-supervised learning, neurocognitive systems can extract structure from their inputs, attempt to predict how the inputs will evolve, and use any discrepancy to improve their representations and computations (cf. (Buckner, forthcoming)). But even self-supervised learning falls short because, in general, self-supervised learning does not involve direct feedback from either the system, the body, or the environment about the effects of the system's actions—if nothing else, because typical artificial neural networks do not act in the world through a body in real time. In contrast, neurocognitive systems are constantly directing their body to act within their environment, use efference copies of their own motor commands to adjust their expectations about how their sensory inputs will change, and collect information about the effects of their motor commands on both body and environment shortly after issuing the commands. Thus, neurocognitive systems can and do use constant, real-time feedback to correct their structure so as to improve their performance.

This lacuna is addressed in part by reinforcement learning. Like artificial systems undergoing reinforcement learning, neurocognitive systems can adjust their action selection by responding to rewards and punishments. There are at least four important differences. First, neurocognitive systems learn in the real world within a relatively short amount of time, whereas current AI techniques are too inefficient to learn realistic tasks in the real world within a reasonable time; learning occurs within simulated worlds and then the acquired knowledge may be transferred to the real world with some degree of success (OpenAI et al., 2019a,b). Second, neurocognitive systems—unlike ordinary artificial neural networks—include an *internal* system of evaluative signals, so neurocognitive systems are not limited to learning from external evaluative signals. Third, neurocognitive systems use several different types of internal reward and punishment signals instead of just one type of evaluative signal. Fourth, insofar as neurocognitive systems can learn from external evaluative signals, such as a parent or teacher telling them “Yes” or “No,” they have to first learn to interpret such signals. To distinguish the type of learning that neurocognitive systems engage in from standard AI techniques, I will call it *active* learning^11^.

The most important feature that active learning shares with AI methods is that *the learning process itself shapes the computations at the same time that it builds the representations*. This marks a critical difference from conventional computers. In conventional computers, the processor manipulates data in accordance with instructions, its circuitry usually remains the same over time, while instructions and data are stored in separate memory registers. Computer instructions have internal semantic content that correspond to the operations performed by the processor, while data can mean anything at all—their content need not have anything to do with the computational operations performed on them. Usually, the operations performed on data match their contents, but this happens only because programmers and users ensure that they do. In fact, computer data need not even mean anything at all. Because of this, if computer data have semantic content at all, as they usually do, they have *derivative* content.

In contrast, in learning neural networks, the operations performed by the units are what activates their representational states, and the representational properties of the states are what allows the network to perform subsequent computational operations efficiently. This mutual dependence exists because both the computational operations and the representations are constructed, jointly and at the same time, by one and the same learning process (cf. Shea, 2018, p. 217). As a result, *within learning neural networks, computations and representations are mutually constitutive of each other and, thus, automatically coordinated*. This is not enough to conclude that neural networks have original, causally efficacious semantic content, but we will soon see that it is an important step in that direction^12^. What is also needed in order to acquire original semantic content is that the network be embodied, embedded, and enactive.

Before getting there, I want to point out another, underappreciated difference between neurocomputational systems and conventional computers. Within conventional computers, the only kind of information processing that takes place is the computation of outputs based on inputs and internal states. In contrast, neurocomputational systems are constantly engaged in two types of information processing at once. Like conventional computers, they yield outputs as a function of their inputs and internal states. *Unlike* conventional computers, they also learn—that is, they use a number of information sources together with their self-organizing capacity to alter their structure and, therefore, their future functions.

It's worth pointing out what sorts of information sources neurocomputational systems can use to actively learn. They include the timing and frequency of their vehicles (primarily, neuronal spikes), the channels through which input signals arrive (visual, auditory, olfactory, etc.), the correlation between one portion of a signal and another portion, and the dependencies between various sorts of input signals (from the environment, body, or neurocognitive system itself, as in the case of efference copy), internal states (such as internal states of the simulations of body and environment and the internal evaluative signals they elicit), and output signals (such as motor commands). By exploiting these relationships and performing operations that are sensitive to them, a neurocognitive system can process information using medium-independent vehicles. In addition, by exploiting the different patterns of dependencies that occur between internal signals and signals from the body, on one hand, and between internal signals and signals from the environment, on the other hand, neurocognitive systems can learn to distinguish between their body and their environment. The upshot is that neurocognitive systems can build representations with original semantic content because neural representations and the computations that manipulate them are functions not only of each single network's inputs and internal states but also of the real-time dependencies between different portions of the whole neurocognitive system's inputs as well as between inputs, internal states, and outputs, which in turn carries information about the body and environment of the system.

Thus, active learning requires *embodiment*—that is, a tight dynamic coupling between neurocognitive system and body^13^. This is true not only because the body contains the sensors and effectors that neurocognitive systems need in order to receive information and act on it. It's also because the real-time feedback loop between neurocognitive systems and their body, whereby the body moves in direct response to neural activity and almost immediately sends information back to the neurocognitive system about how it's moved, is needed for the neurocognitive system to learn how to represent its body, how to represent its body *distinctly* from its environment, and how to effectively simulate and control its body. Since the body is, in turn, the main receiver of information about the environment, the neurocognitive system could not fulfill its learning potential—much less learn how to direct its body within its environment by using internal simulations as a guide—without its constant dynamic interaction with its body.

Active learning requires *embeddedness* as well—that is, a tight dynamic coupling between nervous system, body, and environment. This is true not only because the environment contains the sources of information most senses are sensitive to (except for proprioception, which is perception of the body itself) or because the body itself could not function in the absence of its environment. It's also because the real-time feedback loop between neurocognitive systems and their environment—whereby the environment mostly remains the same regardless of the organism's movements even while the perspective of the organism changes, and yet the environment also changes in specific ways that depend on the actions performed by the organism—is needed for the neurocognitive system to learn how to represent its environment, how to represent its environment *distinctly* from its body, and how to effectively simulate and act within its environment. For example, abnormal visual stimulation during a developmentally critical period impairs vision in ways that can be irreversible (e.g., Hubel and Wiesel, 1970). The neurocognitive system cannot develop properly and cannot fulfill its learning potential without dynamically interacting with its environment, in a way that is mediated by its body.

Active learning requires *enaction* too. For present purposes, enaction is a kind of dynamic interdependence of a system and its environment that unfolds continuously in real time. Specifically, when enaction occurs, cognitive states and processes affect the organism's body and environment while the body and environment affect cognitive states and processes (cf. what Ward et al., 2017 call “sensorimotor enactivism”). Enaction in this sense is already largely implicit in what I said above—let's highlight its most relevant aspects. At any given time, the neurocognitive system is building and updating a simulation of its body and environment and using such a simulation to guide behavior. Meanwhile, each motor command affects (i) how the body moves, (ii) how the sensory input changes (if nothing else, because the position of the body relative to its environment changes), and (iii) some ways that the environment changes (because the organism's actions change it). Moreover, the simulation is attempting to predict how all of this is about to unfold, and the system compares its predictions to its sensory data. Sensory data, in turn, are the main way that the environment affects neurocognitive systems in real time. All of the dependencies between sensory inputs and motor actions are constantly exploited by neurocognitive systems to update their internal simulations as well as to learn how to improve their simulations and the way they guide behavior.

Finally, active learning requires *affect*. In the most basic sense, affect is a system of internal signals that evaluate the state of the organism and its environment to motivate the selection of actions that satisfy the organism's needs. Animals, or at least animals of sufficient complexity and behavioral flexibility, need affect in this sense to select actions that satisfy their needs as well as evaluate external situations and, eventually, learn to select action sequences that are adaptive within different situations. As we have seen, affect in this sense is an aspect of reinforcement learning, which is an aspect of active learning.

As a result of the dependence of active learning on the situatedness of neurocognitive systems, neural representations and computations themselves are embodied, embedded, enactive, and affective. That is, neural representations and computations are the result of the tight interdependence between neurocognitive systems and their body and environment—neurocognitive systems track their targets and guide behavior thanks to their situatedness.

This account is a kind of content externalism, to the effect that neural representations require a direct dynamical coupling to the body and environment in order to exist at all as well as to acquire their semantic content. Content is determined in part by the environment together with the interaction between the nervous system and its environment. As a consequence, neural representations are individuated at least in part by the external variables they have the function to track. This accords with standard definitions of content externalism (Rowlands et al., 2020).

This content externalism is a close relative of but should not be confused with the traditional content externalism defended by Putnam 1975. According to traditional content externalism (adapted to neurocognitive systems), a difference between two environments that is undetectable by the organism, such as a difference in chemical composition between two substances that the organism has no sensory ability to discriminate, is enough to alter the semantic content of a representation. For example, suppose that an organism *A* has learned to activate representations of type *R* in the presence of substance *S* so as to guide behavior with respect to *S*. In light of teleosemantics, tokens of *R* represent *S*. Suppose that organism *A* has an exactly similar duplicate A^*^ who lives in an environment where substance S^*^ is present in exactly the same contexts in which *S* is present within *A*'s environment, yet neither the original organism *A* nor its duplicate A^*^ has any way to distinguish S^*^ from *S*. As a result, within the duplicate A^*^, tokens of *R* get activated in the presence of S^*^. Traditional content externalism maintains that, in the duplicate A^*^, tokens of *R* represent S^*^ rather than *S*.

Traditional content externalism is neither needed nor plausible within the kind of naturalistic perspective I advocate. The sort of case envisioned by traditional content externalism is an exotic case that is unlikely to occur in real life. If it were to occur, the reasonable thing to say is that there are two types of substances, *S* and S^*^, represented by tokens of *R*. A real-world example is the gemstone jade, which may be composed of either of two chemically different minerals, jadeite and nephrite^14^. Prior to modern chemistry, no one knew that there were two types of jade. Nevertheless, then as now, and contrary to traditional content externalism, the term “jade” does not mean just jadeite or just nephrite depending on whether we are looking at jadeite or nephrite, or whether we are in an environment where only jadeite is present or only nephrite is present, or, as traditional content externalists would put it, whether we live on a planet where just jadeite or just nephrite is present. “Jade” just means jade, i.e., something that can be either jadeite or nephrite. By the same token, neural representations represent what neurocognitive systems use them to track, regardless of how many different types of underlying structures activate the same representation^15^.

In conclusion, neural representations emerge diachronically through the dynamical interaction between neurocognitive systems, their body, and their environment, and they depend on such a dynamic interaction for their existence and updating. This situatedness of neural representations allows us to solve the problems of content.

## How Situatedness Solves the Problems of Content

The first problem is the source of original semantic content: how do neural representations acquire original (i.e., non-derivative) semantic content? The situatedness of neurocognitive systems is the very source of their representations' original content. As we've seen, original content itself emerges via a combination of biological evolution and active learning from the constant interaction between nervous system, body, and environment. The original content of a neural representation is a property acquired by the representation via a combination of evolution shaping development and active learning that the system undergoes as it constructs internal simulations of its body and environment to guide the organism's behavior. Perceptual representations and their original content may be more dependent on receiving sensory information than on guiding action, while the reverse may be true of motor representations; nevertheless, for all types of neural representations to be normally acquired and coordinated, all the forms of situatedness we discussed must contribute^16^.

The second problem is the coordination between vehicles and their content: how do vehicles and contents get matched with one another so that the computational operations the nervous system performs over the vehicles match their semantic content? Situatedness solves the coordination problem because the contents themselves are an aspect of the vehicles' functional role, and such a functional role (including the computational operations to be performed on the vehicles) are learned by the system via its interaction with body and environment and the feedback it receives through that interaction at the same time that the content itself is acquired. As we've seen, in neurocognitive systems there is no separation between the semantic content of a neural representation and the computational operations performed over them. The computational operations are an aspect of what gives a neural representation its content; neural representations with the content they have are what allows subsequent computational operations to be performed; the contents and the computational operations are acquired together as the system undergoes active learning.

Let's consider this a bit further. When a neurocognitive system begins to develop, it possesses some ability to process its inputs, build internal states, deliver outputs, and learn from the feedback it receives from itself, its body, and its environment. These initial operations may be partially random but they are also constrained by the architecture and biophysical properties of the system, the morphology and organization of the body, and the structure of the environment. The system may already have a system of internal representations built by developmental processes, or it may be closer to a blank slate. If the system does have innate internal representations it must be because evolutionary and developmental processes sufficiently analogous to active learning have constructed them so that their semantic content matches the operations performed by the system over them or else such representations could not function as such. Over time, it is precisely the process of dynamic interaction between nervous system, body, and environment that allows the system to acquire new or more sophisticated representations at the same time that it learns how to use them. Thus, the neural representational vehicles and their content can only arise together because they are two sides of the same coin. The matching between vehicles and contents is guaranteed by the fact that both contents and the operations performed over the vehicles are joint products of the same active learning process.

The third problem is the causal efficacy of content: how can the semantic content of neural representations be causally efficacious? Situatedness solves the causal efficacy problem because, as we've seen, the content of situated neural representations is an aspect of the causal powers of its vehicles. As a result, unlike typical artificial computing systems, neurocomputational systems are sensitive to the semantic content of their vehicles.

To illustrate, consider a token *r* of neural representation type *R*. Suppose that the system has actively learned to activate tokens of *R* to track and simulate the presence of dogs in its environment and guide behavior with respect to dogs (e.g., Bracci et al., 2019). According to the version of informational teleosemantics I advocate, *r* has original semantic content that can be expressed, approximately, by *dog there now and will likely evolve in such and such a way*. Such a content is not something distinct from and independent of *r*'s causal powers. Rather, *r*'s content is both a manifestation of some of the neurocognitive system's causal powers and a causal power of its own, which can trigger further manifestations. In this case, *r*'s content is an aspect of its power (again, within the context of the neurocognitive system) to guide the system's behavior with respect to a dog being there now. This is made possible by the automatic coordination of *r* (and the computations that process *r*) with *r*'s content that is created when the disposition to activate tokens of *R* is constructed within the system via active learning. Thus, *r*'s content causes the system's behavior with regards to a dog being there now. This is how the semantic content of neural representations causally explains behavior.

The fourth problem is the indeterminacy of content: how can neural representations be said to have semantic content when theorists can't agree on what content they have? Situatedness solves the problem of the indeterminacy of content because the content of the kind of basic neural representations we've been discussing need not have fully determinate semantic content like declarative sentences within a human language. Neural representations have the kind of content that the system needs in order to guide behavior; the kind of content that is ecologically significant and that evolution can act on. The type of behavior depends on the type of organism, and the content of individual neural representations is for neuroscientists to investigate empirically, not for philosophers to intuit about. By investigating the response properties of neurons and neuronal populations, neuroscientists can determine what such neurons or populations are most responsive to under relatively good sensory conditions, and that is their semantic content. If there are different, nonequivalent ways of labeling such contents linguistically (e.g., “fly,” “small dark moving entity,” “food”; or “*S*” versus “S^*^”), this doesn't matter so long as all such labels are extensionally equivalent within the relevant ecological niche^17^. Only when it comes to linguistic cognition do the very special neurolinguistic systems that are involved acquire the kind of categorical contents that admit of full-blown truth conditions. How to get there is a complex story that still needs to be told in detail (some hints are provided in Piccinini and Hetherington, unpublished; Piccinini, 2020b, 2022).

The fifth problem is the distality of content: why should the distal stimulus be the content of a neural representation rather than any of its more proximal stimuli? Situatedness solves the distality problem because different items along the causal chain from distal stimulus to neural representations exhibit different patterns of dependency. As we've seen, neural representations are not static—they dynamically predict the evolution of their target and guide behavior with regards to the target. Meanwhile, the system obtains and processes feedback in response to its actions. At the very least, the organism's movements, including its eye movements, constantly change the precise point of view from which the nervous system obtains sensory data from any given target. The dependency patterns between different items along the causal chain from distal stimulus to internal states are different, the different items evolve in different ways, and changing point of view alters them in different ways. Therefore, as soon as the system attempts to predict how something will evolve over time and improve its predictive performance as well as its action efficacy, it must extract the invariants that are relevant to external stimuli—those it might have to interact with—and discard any invariants that pertain to more proximal stimuli as spurious. It is part and parcel of a neural systems' active learning to acquire representations capable of predicting the evolution of the distal stimuli—those the system might actually interact with^18^.

The sixth and last problem is the possibility of misrepresentation: how can a neural representation misrepresent a stimulus that triggers it? Traditional information teleosemantics is often thought to provide a satisfactory account of misrepresentation. The solution is supposed to be that misrepresentation is failure to perform representational function. This is a huge step in the right direction but it's not enough by itself. The problem with this standard solution is that it requires determining representational function precisely enough to make room for misrepresentation. Specifically, there must be something that determines what each internal state has the function to represent so that, when the state responds to something else, misrepresentation ensues. Extant proposals are that either evolution (Neander, 2017) or an appropriate learning period (Dretske, 1988) determine what each state has the function to represent. I already ruled out evolution as the source of the right notion of function, so that's a nonstarter. As to learning, no one has found a principled way to distinguish the learning period from the rest of the life of a representational state, such that after the learning period is over the representational function is fixed. In some cases, there is a critical learning period that may be the basis for establishing the representational functions of internal states. But, in general, neurocognitive systems never stop learning!

Situatedness comes to the rescue because, again, different stimuli engage in different patterns of dependencies. The most obvious difference is feedback in response to the organism's actions. Again, neural representations are dynamical simulations of their environment, which are largely learned. At any given time, neurocognitive systems have multiple representations that could be activated in response to incoming sensory data. Suppose that, during a dark night, in response to a stimulus, a system activates a COW-representation—that is, the kind of representation it has learned to activate when it needs to simulate cows. The COW-representation yields specific predictions about how the sensory data will change if the stimulus is approached (i.e., it will look more and more distinctly like a cow), or if the stimulus makes a vocalization (i.e., it will “moo”), or what their footprints will look like, and so forth. As soon as enough sensory feedback is collected that matches a different representation better than the current one, the system itself should self-correct, and it will self-correct if it's functioning properly. That is, the system will deactivate the COW-representation and activate one that fits the sensory data better—e.g., a HORSE-representation. Thus, misrepresentation occurs when a system activates a representation, targeting a stimulus, which makes worse predictions about incoming data about what a stimulus will do and how it will appear under various possible conditions than an alternate representation that is also available to the system. In short, misrepresentation arises from the interaction of learning, simulation, and the ability to detect errors and make corrections. The ability of neurocognitive systems to correct their own misrepresentations is also another way of seeing that their content is causally efficacious (Bielecka and Miłkowski, 2020)^19^.

## Conclusion

I have argued that, far from being opposites as so many have thought, situatedness and representation are more deeply intertwined than anyone suspected. What makes neural representations possible is the very situatedness of the processes that acquire neural computations and representations.

Neurocognitive systems are indeed embodied, embedded, affective, dynamically interact with their environment, and use feedback from their interaction to acquire their own representations and computations via active learning. This accounts for the following: (1) neurocognitive systems construct neural representations with original semantic content, (2) their neural vehicles and the way they are processed are automatically coordinated with their content, (3) such content is a special kind of causal power and hence causally efficacious, (4) is determinate enough for the system's purposes, (5) represents the distal stimulus, and (6) can misrepresent. This proposal hints at what artifacts should be like in order to acquire the basic cognitive abilities possessed by neurocognitive systems.

## Data Availability Statement

The original contributions presented in the study are included in the article further inquiries can be directed to the corresponding author.

## Author Contributions

All authors listed have made a substantial, direct, and intellectual contribution to the work and approved it for publication.

## Conflict of Interest

The author declares that the research was conducted in the absence of any commercial or financial relationships that could be construed as a potential conflict of interest.

## Publisher's Note

All claims expressed in this article are solely those of the authors and do not necessarily represent those of their affiliated organizations, or those of the publisher, the editors and the reviewers. Any product that may be evaluated in this article, or claim that may be made by its manufacturer, is not guaranteed or endorsed by the publisher.

## Footnotes

^1^This self-organizing feature of organisms has long been emphasized by what is sometimes called *autopoietic enactivism* (Varela et al., 1974; Ward et al., 2017).

^2^Some complex organisms have sentience and sapience, which give rise to nonbiological functions, which are stable contributions to nonbiological goals. Nonbiological functions are not especially relevant here.

^3^Roughly, digital computations can operate over sequences of discrete states, analog computations can operate over continuous variables, and neural computations operate over spike trains; within spike trains, spikes are distinct from one another, which makes them somewhat similar to discrete states, but their frequency and sometimes their timing are functionally significant, which makes them somewhat similar to continuous variables. Since the vehicles of neural computation shares similarities and differences with the vehicles of both digital and analog computation, neural computation is sui generis. A more detailed treatment is in Piccinini (2020a, especially Chs. 6 and 13).

^4^The following account of neural representation and its content was influenced most directly by Piccinini (2020a, Ch. 12), which is a descendant of Thomson and Piccinini (2018), and by Lee (2021). Other important recent sources that influenced me on the problems of content and related matters include Gładziejewski (2015); Ramsey (2016); Gładziejewski and Miłkowski (2017); Miłkowski (2017); Neander (2017); Buckner (2018, forthcoming); Dewhurst and Villalobos (2018); Lee (2018); Shea (2018); Millikan (2021); Poldrack (2021), and Bielecka and Miłkowski (2020).

^5^Some authors prefer the notion of similarity to that of homomorphism. For a recent account of semantic information carried by structural representations in terms of similarity, see Miłkowski 2021.

^6^The notion of representation primarily under discussion here is that of *indicative* representation, whose function is to fit the world. There are also *imperative* representations, whose function is to change the world to fit them. I discuss imperative representations in Piccinini (2020a, Ch. 12).

^7^Facchin 2021 has recently questioned whether structural representations' functional role is genuinely representational. Roughly, he argues that (a) receptors are not genuinely representational, yet (b) some receptors are structural representations; therefore, (c) some structural representations are not genuinely representational. Even as Facchin presents his argument, it leaves room for *some* structural representations to be genuinely representational, which is all I need. In any case, Facchin does not establish (a); instead, he makes a plausible case that (a') *some* receptors are not genuinely representational. Needless to say, (c) doesn't follow from (a') and (b).

^8^For an account that goes somewhat in the same direction, see Shea (2018, p. 10, Chs. 3 and 4).

^9^My version of teleosemantics is not based on the usual, selectionist account of functions, according to which functions are selected effects (e.g., Neander, 2017); it is based on the goal-contribution account of function I briefly reviewed in the previous section according to which functions are stable contributions to the goals of organisms. One advantage of this innovation is that it makes it possible for semantic content to be causally efficacious.

^10^Shea (2018, p. 36, 39) has independently argued that content arises out of a combination of a vehicle's intrinsic and relational properties.

^11^The label “active learning” is used in pedagogy for a method of learning in which students are not merely listening to lectures or reading material (i.e., passive learning) but are actively engaged with the material through discussions, writing assignments, role play, etc. I am repurposing this label for the type of learning that neurocognitive systems spontaneously engage in.

^12^For an independently developed yet converging argument that the coordination between vehicles and their content helps solve the problem of the causal efficacy of content see Shea (unpublished).

^13^Different authors characterize embodiment, embeddedness, and enaction in different ways (for a recent review, see Shapiro and Spaulding, 2021). Some authors define embodiment, embeddedness, or enaction in ways that preclude computation and representation (e.g., Thompson, 2007, p. 13); this begs the question at hand. I adopt characterizations that are present in the literature, do not beg the question of computation and representation, and suit present purposes. A fuller treatment of the relation between the present argument and the many themes from the literature on situated cognition will have to wait for another occasion.

^14^Jade was discussed extensively in the debate on reductionism about mental states (Kim, 1992; Fodor, 1997). I am putting the example to a different use.

^15^This conclusion is consistent with many critiques of traditional content externalism; see Sections 3.2 and 3.3 of Rowlands et al. 2020 for a review.

^16^Thanks to a referee for pointing out that there may be cases of atypical development, neurodiversity, or neuropathology in which some forms of situatedness do not contribute to the development of neural representations.

^17^Whether the labels in our frog example are actually extensionally equivalent within the ecological niche of frogs is questionable. For instance, frogs eat way more than just flies, so “fly” is not extensionally equivalent to “food”. At any rate, we should let neuroscientists find the best way to characterize the content of neural representations.

^18^Some recent teleosemantic literature moves at least part of the way in the same direction (e.g., Neander, 2017, Ch. 9; Garson, 2019; Schulte, 2021).

^19^On the role of error correction, I'm also indebted to Gładziejewski (2015).
